# Long-lasting effects on cognition and mental health in patients with post COVID-19 condition following a mild SARS-CoV-2 infection: A longitudinal case–control study

**DOI:** 10.1192/j.eurpsy.2025.10108

**Published:** 2025-09-12

**Authors:** Goran Papenberg, Marika C. Möller, Gabriela Markovic, Kristian Borg, Jonas Stenberg, Stina Hedström, Frida Smids, Emil Norrman, Andrea Aejmelaeus-Lindström, Grégoria Kalpouzos, Erika J. Laukka

**Affiliations:** 1 Aging Research Center, Department of Neurobiology, Care Sciences and Society, Karolinska Institutet and Stockholm University, Stockholm, Sweden; 2 Department of Clinical Sciences, Danderyd Hospital, Karolinska Institutet, Stockholm, Sweden; 3 Department of Rehabilitation Medicine, Danderyd University Hospital, Stockholm, Sweden; 4 Department of Clinical Neuroscience, Karolinska Institutet, Stockholm, Sweden; 5 Stockholm Gerontology Research Center, Stockholm, Sweden

**Keywords:** anxiety, cognition, depression, fatigue, mental health, mild post COVID-19 condition, PCC

## Abstract

**Background:**

The prevalence of prolonged symptoms following a severe acute respiratory syndrome coronavirus 2 (SARS-CoV-2) infection represents a significant health challenge with potentially severe individual and societal costs. Our study investigates the long-term cognitive and mental health consequences associated with post-coronavirus disease 2019 (COVID-19) condition (PCC) following a mild SARS-CoV-2 infection.

**Methods:**

We conducted longitudinal assessments of cognitive performance and mental health in 50 post-COVID-19 patients and 48 matched healthy controls across 10 months, starting on average 2 years after infection. Cognitive function was evaluated using a comprehensive neuropsychological battery of standardized tests, while mental health was assessed via self-reported questionnaires. Data were analyzed with linear mixed models.

**Results:**

Initial group differences in cognitive performance were observed for memory, executive functioning, and perceptual speed, with worse performance in patients. Improvement across the follow-up period occurred for most tasks, with PCC patients displaying greater improvement compared to healthy controls for some memory and executive function tasks, reaching performance levels of the control group. Fatigue and mental health measures remained elevated in the patient group, with worsening in general fatigue and a small improvement in fatigue after cognitive testing. Factors such as male sex, absence of burnout history, and lower depression scores at baseline predicted cognitive recovery in the patient group.

**Conclusions:**

Our study underscores the importance of addressing cognitive and psychological effects following mild SARS-CoV-2 infection, as persistent fatigue, low mental health, and cognitive impairments significantly impact individuals’ ability to return to their pre-COVID professional and personal lives.

## Introduction

The large number of individuals with long-lasting symptoms following a coronavirus disease 2019 (COVID-19) is a serious health problem with potential severe consequences for affected individuals and increased strain on the healthcare system. The current study investigates the impact of a post-COVID-19 condition (PCC) following a mild severe acute respiratory syndrome coronavirus 2 (SARS-CoV-2) infection on long-lasting cognitive deficits and mental health symptomatology.

PCC is defined as having a history of probable or confirmed SARS-CoV-2 infection, usually 3 months from the onset of COVID-19, with symptoms that last for at least 2 months and are not explained by an alternative diagnosis (WHO, 2021) [1]. Common symptoms include fatigue, depression, anxiety, shortness of breath, and cognitive dysfunction [2–5]. Among persons who experienced an initially mild SARS-CoV-2 infection, many have remaining symptoms after recovery that affect their daily abilities, with a high burden of anxiety and depression, as well as memory impairment, based on meta-analytic evidence [3]. Early data showed that 1 in 10 healthcare workers experienced long-term effects 8 months after mild COVID-19, with at least one moderate to severe symptom (e.g., fatigue and memory impairment), negatively impacting their work, social, or home life [6]. More recently, a study reported attention and memory impairments following mild COVID-19 after 8 months of first symptoms, as compared to a control group [7]. Other studies observed global cognitive impairments in mild COVID-19 patients [8–10], with assessments performed between 8 and 18 months after infection. A rare longitudinal study, which followed the participants across 6 months, reported primarily persistent impairment in perceptual speed in PCC as compared to controls [11]. Initial assessments of PCC patients were performed on average 13 months after the infection, which points to long-term deficits in this group. That said, the sample included PCC patients who were hospitalized (including intensive care), which makes it difficult to conclude mild COVID-19. Another longitudinal study concluded that many PCC patients tested 7 months after infection still have cognitive symptoms relative to norms after 13 months, with some improvements in memory [12]. Further, depression, anxiety, and fatigue have been emphasized as major sequelae in long COVID-19 syndrome [13, 14], which may influence recovery. So far, however, longitudinal studies involving repeated neuropsychological testing in both PCC with mild COVID-19 and healthy controls are scarce.

We performed longitudinal investigations to gain an understanding of (a) the nature, severity, and duration of long-term effects of mild COVID-19 on cognitive function and mental health, and (b) which individuals are more likely to recover from long-term cognitive deficits. Previously, we reported that patients with post-COVID-19 with an initially mild infection exhibited general cognitive deficits compared to standardized norms [8]. In the present study, we followed up a subsample of these individuals for an average of 10 months and compared their performance changes with a matched control group.

## Methods

This is a case–control longitudinal study with data prospectively collected at two timepoints. The sample consisted of PCC patients (*n* = 50) and healthy controls (*n* = 48) who were followed across an average of 10 months (Table 1). All patients had a confirmed PCC diagnosis (according to WHO criteria, 2021) and had experienced a SARS-CoV-2 infection (January 2020 to February 2021) between 8 and 35 months (mean = 23 months) before the first cognitive testing (March 2021 to February 2023). Patients were recruited from the Cognitive Post COVID-19 Clinic at Danderyd University Hospital in Stockholm, Sweden, who sought health care because of self-experienced cognitive problems. All had an initially mild infection (i.e., most were not admitted to the hospital, and none were admitted to intensive care). In addition, we recruited a control group of individuals who contracted COVID-19 during the same period (February 2020 to January 2021) as the patient group but who did not experience cognitive deficits after recovery (first SARS-CoV-2 infection on average 25 months before first cognitive testing, with a range of 12–35 months). The control group was recruited through advertisement via the Karolinska Institute webpage and Facebook. The patients underwent their first assessment at Danderyd University Hospital, whereas the controls performed both testing occasions at our research center. Patients and controls were well-matched on several demographic variables (Table 1), although the patient group had slightly fewer years of education and longer follow-up time. In addition, patients had a history of depression and burnout more frequently based on self-reports. While all patients were assessed at both baseline and follow-up, seven controls dropped out at different stages of the follow-up assessment (i.e., did not reply at the interview stage, did not show up, or canceled).

**Table 1.** Demographic information across groups

	Patients	Controls
*n*	50	48
Sex, % female	70	65^a^
Age at baseline, *M* (SD)	48 (10)	50 (10)^b^
Swedish, native speaker, %	88	90^a^
Education, *M* (SD)	17 (3)	18 (2)^c^
Vaccinated, %	84	94^a^
WAIS information test, *M* (SD)	19 (4)^d^	20 (3)^b^
WAIS matrices, *M* (SD)	21 (3)	22 (3)^b^
Days between baseline and follow-up, *M* (SD)	322 (98)	269 (1)^e^
History of depression, %	28	8^f^
History of burnout, %	34	15^g^

a Chi-square test = not significant.

b Univariate analysis of variance = not significant.

c Univariate analysis of variance: *F*(1,96) = 9.3, *p* = .003.

d *n* = 48.

e Univariate analysis of variance: *F*(1,89) = 9.8, *p* = .002 (*n* = 41 for controls).

f Chi-square test: *χ* ^2^ (1, *n* = 95) = 5.6, *p* < .05 (3 missing values).

g Chi-square test: *χ* ^2^ (1, *n* = 97) = 4.8, *p* < .05 (1 missing values).

The study was approved by the Swedish Ethical Review Authority. All participants provided written informed consent. The authors assert that all procedures contributing to this work comply with the ethical standards of the relevant national and institutional committees on human experimentation and with the Helsinki Declaration of 1975, as revised in 2008.

## Cognitive battery

The assessment was administered by neuropsychologists within the clinical setting. The control group was assessed by trained research assistants and psychologists. All assessments followed a predefined protocol across both groups. The cognitive battery consisted of standardized tests, typically used in clinical settings, covering a range of cognitive domains. Because our patient group was highly educated (Table 1), we focus on tasks and conditions with a high cognitive load (see Supplementary Materials for details). To make estimates comparable across domains, all cognitive scores were standardized using the baseline mean and standard deviation (SD) of the total sample as the standardization base. Higher scores always indicated better performance, except when performance was assessed in time. At baseline, the Wechsler Adult Intelligence Scale, fourth edition (WAIS-IV), Information and Matrices were performed as measures of participants’ premorbid cognitive level. The Information subtest aimed to test general knowledge, whereas Matrices is a nonverbal reasoning task in which individuals are asked to identify patterns in abstract designs. Furthermore, the following tasks were assessed longitudinally: *Buschke’s selective reminding task* (*SRT*), *Color-Word Interference Test* (*CWIT*), D-KEFS *Trail Making Test*, D-KEFS Verbal Fluency, and *Ruff 2&7 selective attention test* (*Ruff 2&7*). We included two tests from WAIS-IV, which were administered according to the manual: *Digit Symbol* (*Coding*) and *Digit Span* (*see Supplementary Materials for detailed description of tasks and variables used*).

## Self-rated fatigue and mental health

In addition to measures of fatigue, participants filled out several questionnaires related to their well-being in relation to the testing session (*see Supplementary Materials for detailed descriptions of questionnaires*). More specifically, the following questionnaires were used to assess fatigue and mental health: *Fatigue Visual Analogue Scale* (*VAS*), *Multidimensional Fatigue Inventory 20* (*MFI-20*), and *Hospital Anxiety Depression Scale* (HADS).

## Statistical analyses

To investigate the effects of post-COVID-19 on cognitive and mental health changes across time, we conducted linear mixed models in STATA (SE 18.5). Fixed effects included group (patients and healthy controls), time (baseline and follow-up), and an interaction term between group and time. To document changes in cognition and mental health, we first run models with time only. Random intercept and slope were estimated as the random part in the mixed-effect model. We used unstructured variance–covariance matrices for all models. Missing data were handled with the maximum likelihood method. All linear mixed model analyses were adjusted for age at baseline, sex, as well as years of education. Education and age were centered at the sample mean. Follow-up time (differences between baseline and follow-up) was used as an additional mean-centered covariate to control for different time intervals in the longitudinal analyses. Predicted means were estimated with the “margins” command in STATA for illustration in graphs, which were created with R (*ggplot* function).

Furthermore, we explored which interindividual difference factors were associated with changes in cognition by conducting correlational analyses (Spearman’s *r*) between predictors of interest and significant changes in patients (follow-up minus baseline). The following potential predictors were considered: age, sex, education, history of depression and burnout, and HADS scores (depression and anxiety), VAS scores (before and after cognitive testing), and MFI scores (general fatigue, physical fatigue, and mental fatigue) at baseline. Values greater or smaller than 4 SDs from the mean were excluded from the analyses and set as missing values. The *p*-value for statistical significance was set to 0.05.

## Results

### Longitudinal changes in cognition and mental health independent of group

Raw data of the variables of interest are presented in the Supplementary Materials (Table S1). Analyses revealed significant improvements in eight cognitive measures (see Table 2), while three measures showed no significant changes across time. Regarding self-assessments, VAS-before testing showed no change, but VAS-after testing improved over time (see Table 2). Significant longitudinal changes were further observed for MFI-General Fatigue (worsened), and HADS-Anxiety and HADS-Depression (improved).

**Table 2.** Results of linear mixed models for analyses investigating the effect of time on the cognitive and mental health measures

	Time	
	Coefficient95% CI	*p*-value
Cognition		
Buschke’s selective reminding task		
Total recall	.34 [.22 .47]	<.001
Delayed recall	.38 [.22 .53]	<.001
Color-word interference test		
Interference (time)	−.28 [−.41–.15]	<.001
Switching (time)	−.25 [−.34–.15]	<.001
Trail making test		
Condition 4: switching	−.26 [.38 .13]	<.001
Verbal fluency		
Condition 1: phonetic	.12 [.01 .22]	.034
Condition 2: semantic	.03 [−.07 .14]	.559
Condition 3: switching	.02 [−.15 .20]	.795
Ruff 2&7		
Automatic detection & controlled search speed	.06 [−.03 .17]	.224
Digit symbol (coding)	.30 [.17 .43]	<.001
Digit span (backwards)	.21 [04. 37]	.014
Questionnaires		
VAS		
Before cognitive testing	−.08 [−.30 .11]	.396
After cognitive testing	−.22 [−.37–.06]	.006
MFI		
General fatigue	.21 [.01 .40]	.042
Physical fatigue	.04 [−.11 .20]	.597
Mental fatigue	.00 [−.13 .13]	.990
HADS		
Anxiety	−.19 [−.35–.04]	.015
Depression	−.15 [−.30–.00]	.049

Abbreviations: HADS, Hospital Anxiety and Depression Scale; MFI, Multidimensional Fatigue Inventory; VAS, Visual Analogue Tiredness Scale.

### Baseline differences in cognition and changes across time as a function of group

**Figure 1.**
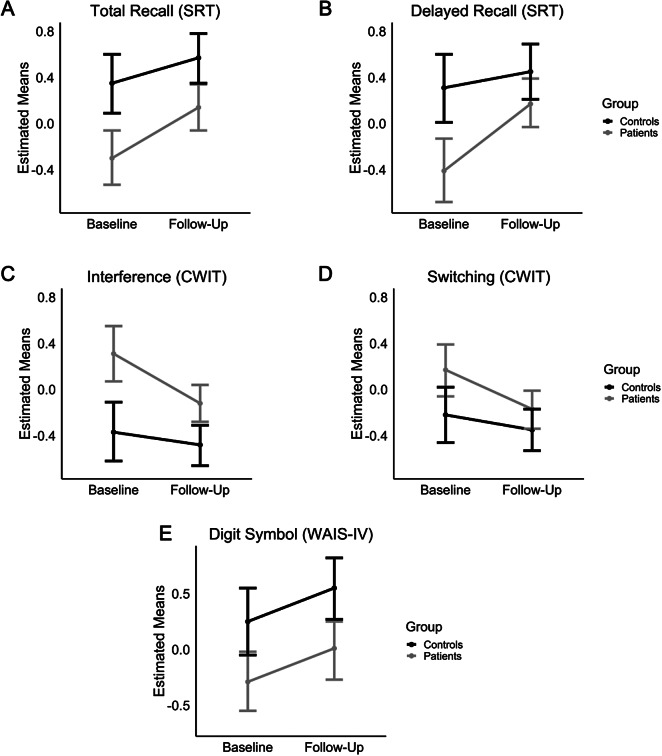
Estimated means based on linear mixed models for (A) Total Recall (SRT), (B) Delayed Recall (SRT), (C) Interference (CWIT, in seconds), (D) Switching (CWIT, in seconds), and (E) Digit Symbol (WAIS-IV) as a function of time (baseline and follow-up) and group (controls and patients). Error bars indicate 95% confidence intervals.

Table 3 summarizes group differences at baseline and the group × time interactions. Significant group differences were observed at baseline for episodic memory, where patients performed worse than controls in the two conditions (Total and Delayed Recall) from the SRT (Figure 1A,B). The interaction with time did not reach conventional significance for Total Recall. By contrast, the interaction between time and group was significant for Delayed Recall, indicating a larger improvement in patients across the follow-up period. At follow-up, the performance of the patients on the Delayed Recall task did not differ from the baseline performance of the healthy controls (*b* = −.13, 95% confidence interval [CI] = [−.51, .25], *p* = .499). Furthermore, patients performed at similar levels as healthy controls at follow-up (*b* = −.27, 95% CI = [−.61, .06], *p* = .112), indicating that the patients have improved to normal range levels.

**Table 3.** Results of linear mixed models for analyses involving the cognitive measures

			Baseline		Interaction	
			Coefficient	*p*-value	Coefficient	*p*-value
Cognition	*n* patients	*n* controls				
	baseline/follow-up	baseline/follow-up				
Buschke’s selective reminding task						
Total recall	46/49	48/41	−.65 [1.00–.29]	<.001	.22 [−.02 .47]	.079
Delayed recall	47/48	48/41	−.72 [−1.10. –30]	<.001	.44 [.14 .74]	.004
Color-word interference test						
Interference (time)	46/49	48/41	.68 [.32 1.04]	<.001	−.32 [−.58–.06]	.017
Switching (time)	46/49	48/41	.38 [.04 .73]	.029	−.21 [−.40–.01]	.037
Trail making test						
Condition 4: switching	47/50	48/41	.03 [−.27 .33]	.831	−.04 [.29 .21]	.751
Verbal fluency						
Condition 1: phonetic	50/50	48/41	−.01 [.42 .41]	.979	−.06 [−.27 .16]	.596
Condition 2: semantic	50/49	48/41	−.30 [−.71 .11]	.153	.10 [−.11 .31]	.366
Condition 3: switching	50/49	48/41	.26 [−.16 .68]	.221	.02 [−.34 .37]	.927
Ruff 2&7						
Automatic detection & controlled search speed	50/50	48/41	−.02 [−.32 .27]	.884	.08 [−.13 .28]	.447
Digit symbol (coding)	50/48	48/41	−.54 [−.95–.12]	.012	.00 [−.26 .26]	.998
Digit span (backwards)	50/50	48/41	−.18 [−.59 .24]	.403	−.00 [−.34 .33]	.984

*Note*: Baseline coefficients indicate group differences at the first time point. Significant interactions indicate differences in longitudinal changes as a function of group.

Similarly, patients performed worse than healthy controls on the two variables from the CWIT at baseline (Figure 1C,D). For both conditions, the interaction with time was significant, indicating a larger improvement in patients. Again, patients’ performance at follow-up did not differ from the baseline performance of healthy controls (Interference: *b* = .25, 95% CI = [−.06, .56], *p* = .121; Switching: *b* = .04, 95% CI = [−.26, .35], *p* = .788). When comparing the two groups at follow-up, patients performed worse than the control group for Interference (*b* = .36, 95% CI = [−.51, –.25], *p* = .005), while differences were not significant for the Switching condition (*b* = .18, 95% CI = [−.08, .44], *p* = .177). Finally, a group difference at baseline was evident for Digit Symbol Coding (Figure 1E), with patients exhibiting slower performance (*b* = −.54, 95% CI = [−.95, –.12], *p* = .012), with no significant interaction effects with time. These group difference remained significant at follow-up (*b* = −.54, 95% CI = [−.92, –.15], *p* = .006). There were no significant differences or interactions with respect to Ruff 2&7 composite score (see Table S2 for subscales), Verbal Fluency, Trail-Making Test, and Digit Span (see Table 3).

### Baseline differences in mental health and changes across time as a function of group

As compared to healthy controls, patients consistently scored worse on the self-rated measures, such as MFI, HADS, and VAS (see Tables 4 and S1). A significant group × time interaction for the MFI-General Fatigue indicated that self-rated fatigue had worsened in patients over time (*b* = .44, 95% CI = [.18, .69], *p* = .001), while there was no change in healthy controls (*b* = −.05, 95% CI [−.34, .23], *p* = .712). By contrast, a significant group × time interaction with respect to VAS after cognitive testing indicated that patients’ self-rated fatigue after cognitive testing was lower at follow-up but remained higher than for healthy controls at follow-up (*b* = .88, 95% CI [−.54, 1.22], *p* < .001).

**Table 4.** Results of linear mixed models for analyses involving the self-rated mental health scores

			Baseline		Interaction	
			Coefficient	*p*-value	Coefficient	*p*-value
Questionnaires	*n* patients	*n* controls				
	baseline/follow-up	baseline/follow-up				
VAS						
Before cognitive testing	49/50	48/41	.92 [.55 1.30]	<.001	−.19 [−.58 .20]	.348
After cognitive testing	47/50	48/41	1.24 [.92 1.54]	<.001	−.36 [−.65–.06]	.018
MFI						
General fatigue	50/47	47/40	.79 [.41 1.16]	<.001	.49 [.11 .87]	.012
Physical fatigue	50/47	47/40	1.21 [.88 1.54]	<.001	.15 [−.16 .46]	.335
Mental fatigue	50/46	47/40	1.21 [.88 1.54]	<.001	−.01 [−.28 .25]	.932
HADS						
Anxiety	48/47	46/39	.97 [.58 1.38]	<.001	−.22 [−.53 .09]	.170
Depression	48/47	47/39	1.05 [.68 1.43]	<.001	−.19 [−.48 .11]	.208

Abbreviations: HADS, Hospital Anxiety and Depression Scale; MFI, Multidimensional Fatigue Inventory; VAS, Visual Analogue Tiredness Scale.

*Note:* Baseline coefficients indicate group differences at the first time point. Significant interactions indicate differences in longitudinal changes as a function of group.

### Factors associated with cognitive improvements in post-Covid-19 patients

We explored which demographic and individual difference factors were associated with improvements in cognition in patients, focusing on the significant findings reported above for SRT (Delayed Recall) and CWIT (Interference and Switching). Table S3 in the Supplementary Materials shows all associations. Male sex was significantly related to improvements in CWIT-Interference only (*r*_S_ = .31, 95% CI [.16, .47], *p* = .039). Further, patients with no history of burnout were also more likely to improve on this measure (*r*_S_ = .37, 95% CI [.03, .61], *p* = .012). Finally, a lower baseline score on the HADS-Depression scale, indicating fewer depressive symptoms, was related to higher improvement in CWIT-Switching (*r*_S_ = −.34, 95% CI [−.60, .01], *p* = .025).

## Discussion

We performed longitudinal investigations to gain an understanding of the nature, severity, and duration of long-term effects of mild COVID-19 on cognitive function and mental health. We also investigated factors related to better recovery from long-term cognitive deficits. Our study provides important insights into the long-term cognitive deficits following a mild SARS-CoV-2 infection. As compared to a healthy control group, we observed significant cognitive differences in PCC patients with respect to long-term memory, executive functioning, and processing speed at baseline, taking place on average 2 years after the initial infection. Notably, improvements in patients with PCC were observed over the follow-up period in both episodic memory and executive functioning, but not in processing speed. Despite observed improvements in cognitive performance levels, the patient group continued to score at a lower level than the controls for some tasks. Moreover, patients with PCC were characterized by severe self-reported multi-domain fatigue at both baseline and follow-up. While minor improvements were evident in terms of self-rated testing-related fatigue, general fatigue even increased after follow-up for patients, suggesting that their symptoms still made cognitive tasks and everyday functioning effortful. Given the overall pattern, the small improvements in testing-related (state) fatigue may be related to variation associated with patients being tested on a good day or repeated exposure to the same test situation, which may feel less effortful. Relatedly, anxiety and depression scores remained at higher levels compared to controls. Follow-up analyses revealed that demographic and individual difference factors, such as male sex and absence of burnout history or lower depressive symptoms at baseline, were associated with larger cognitive improvements in the patient group. That said, patterns were not consistent across tasks.

Previous studies investigating persistent cognitive deficits in post-COVID patients often yielded mixed results, which may be due to differences in study designs. Studies evaluating post-COVID-19 individuals after mild infections with no control group typically observed global cognitive impairments as compared to standardized norms [8, 9]. Cross-sectional studies that involved mixed samples of post-COVID patients (intensive care unit [ICU], hospitalized, and mild) tested after 11 months of infection, reported global cognitive deficits across different domains [5, 15]. However, when comparing different groups, most cognitive differences were found between ICU patients and healthy controls [15]. More recently, a study reported attention and memory impairments following mild COVID-19 as compared to a control group [7]. Another study did not find any cognitive differences between patients with mild–moderate COVID-19 and healthy controls, assessed 4 months after infection. Instead, the groups differed in anxiety, depression, and stress [16], in line with the group differences in mental health observed in our study. Importantly, a longitudinal study involving a direct longitudinal comparison between mild PCC and a control group indicated persistent cognitive deficits, particularly with respect to speed, after 6 months of follow-up [11]. This pattern was replicated in another study [17]. These findings are in line with our data with respect to speed, the only cognitive domain that did not improve in patients. Although no control group was assessed, another longitudinal study documented memory improvements in PCC patients tested at 7 and 13 months after infection [12]. Other cognitive deficits, including speed and fluency, were still present.

A few individual difference factors were previously found to be related to improvements in cognition, a pattern also observed in our study. This pattern may be due to the very long baseline assessments after the initial infection, which is nearly 2 years in our study. Female sex has been shown to be a risk factor for the development of PCC [18], but male sex was previously reported to be a predictor of cognitive non-recovery [19]. By contrast, the influence of sex on recovery was in the opposite direction in our data. The latter study also reported that patients with depressive symptoms at baseline were less likely to recover from fatigue but did not observe any effect on cognitive non-recovery. Recently, attempts have been made to cluster COVID-19 phenotypes into distinct groups, identifying one group with normal cognitive function, another group with impairments in executive function, and a third group with impairment in memory and speed [20]. The 6-month recovery was worst for the latter group with no objective improvement, which maps onto our finding of memory (Total Recall from SRT) and speed deficits (Digit Symbol) persisting over time. Taken together, our findings suggest that most cognitive deficits improve with time, which may explain the mixed findings in the literature. Cognitive non-recovery in our data was either related to known risk factors or psychiatric symptoms in long-COVID patients, such as female sex and depressive symptoms [21].

The most parsimonious explanation of the pattern of results is that a SARS-CoV-2 infection may impair hippocampal neurogenesis, the formation of new neurons, leading to memory impairments, as well as symptoms of depression and anxiety. In animals, impairment in hippocampal neurogenesis was related to impairment of memory [22], cognitive flexibility [23], as well as depressive and anxiety symptoms [24, 25]. A recent review emphasized poor hippocampal integrity in COVID-19 patients as compared to healthy controls [26], including impairments in neurogenesis and lower volume. Importantly, even mild SARS-CoV-2 infection was associated with lower hippocampal neurogenesis in mice, which was not found with other respiratory viral infections [27]. The latter study also reported decreased oligodendrocytes and myelin loss after mild SARS-CoV-2 infection, which is in line with the observation of persistent white-matter changes in recovered COVID-19 patients as compared to controls [28, 29] and our data on group differences in processing speed, a cognitive domain dependent on the integrity of white-matter tracts [30].

Notably, the patients in our study were characterized by a higher frequency of self-reported history of depression and burnout as compared to controls, suggesting that these may be risk factors for PCC. It has been hypothesized that stress-related suppression of adult hippocampal neurogenesis may be an underlying neurobiological mechanism for the development of burnout [31]. Infection with SARS-CoV-2 may further impair neurogenesis, exacerbating depressive and anxiety-like symptoms and resulting in temporary cognitive impairments.

Major strengths of our study include a comprehensive longitudinal neuropsychological assessment and battery of self-reported questionnaires, as well as a well-matched control group, which was also assessed twice. Some limitations should also be acknowledged. One limitation of our study is the potential impact of self-selection effects, where patients with long-term post-COVID-19 that contacted the rehabilitation clinic may differ systematically from those who did not. It is conceivable that only patients who could physically and psychologically undergo cognitive testing were assessed. Moreover, the patient group was more likely to reschedule their appointment for the follow-up testing due to poor health and fatigue. This may have led to an underestimation of their cognitive deficits. Also, as manifested by the high fatigue score after cognitive testing, the observed cognitive scores of our well-educated patient group may not capture day-to-day fluctuations in their cognitive performance. We have limited information on what kind of activities and treatments the patients took part in between the two testing occasions and how this may have influenced their performance. Some of the cognitive assessments utilized in this study (e.g., Digit Span) may further lack sufficient difficulty for participants with higher educational attainment and complex professional backgrounds. Finally, deficits did not always generalize across all tasks of the same domain, possibly due to a lack of power due to the small sample size.

Taken together, we show that the PCC patients performed worse in memory, executive functioning, and speed, as compared to healthy controls. The patients showed larger improvements over time compared to controls, which in some cases were large enough to revert the patients to normal cognitive levels. These patterns suggest that patients can recover from cognitive impairment even after an extended period following the initial infection.

Additionally, negative outcomes in self-rated mental health and fatigue were observed among patients, with a general lack of improvement over time. Despite some reduction in fatigue after cognitive testing, this score remained higher than in the healthy controls. Together, the partial impairments in cognition and persistent impairments in mental health and fatigue have important implications for individuals’ ability to resume pre-COVID professional and personal activities, emphasizing the need for targeted rehabilitation programs.

## Supporting information

10.1192/j.eurpsy.2025.10108.sm001 Papenberg et al. supplementary material Papenberg et al. supplementary material

## Data Availability

The conditions of our ethics approval do not permit public archiving of pseudoanonymized study data. Researchers seeking access to the data should contact the senior author, Erikka J. Laukka. Access will be granted to named individuals in accordance with ethical procedures governing the reuse of sensitive data. Specifically, requestors must meet the following conditions to obtain the data: completion of a formal data sharing agreement and ethical approval.

## Supplementary material

The supplementary material for this article can be found at http://doi.org/10.1192/j.eurpsy.2025.10108.
