# Supplementary material for: Long-lasting effects on cognition and mental health in patients with post COVID-19 condition following a mild SARS-CoV-2 infection: A longitudinal case–control study
Source: Eur Psychiatry. 2025 Sep 12;68(1):e141. doi: 10.1192/j.eurpsy.2025.10108 (PMC12538188; doi:10.1192/j.eurpsy.2025.10108)
Supplement: Papenberg et al. supplementary material [file S0924933825101089sup001.docx]

**Methods**

**Cognitive Battery**

*Buschke’s selective reminding task (SRT):* This test was used as a measure of episodic memory [1]. The test leader orally presented 12 unrelated words, and the participant was asked to recall the words in no predefined order. The test leader then repeated the words that the participant had not immediately recalled, and the participant was asked to recall all 12 words once more. This procedure was repeated until the participant had recalled all words in two consecutive trials or after a maximum of 12 trials. After a 30-minute delay, the participant was again asked to recall the list of words, without any reminders. Two versions of the test were used, and participants received a different version at the follow-up assessment. The total number of successfully recalled words during the initial 12 trials (max 144) and the number of successfully recalled words after the 30-minute delay (max 12) were used as outcome variables.

Three tests from the Delis-Kaplan Executive Function System (D-KEFS) were administered according to the procedure described in the manual [2].

*Color-Word Interference Test (CWIT):* This test was included as a measure of executive function. It is divided into four conditions, and the third and fourth condition were used as outcome variables in the current study. In the third condition, participants were shown a page with color names printed in incongruent colors (Interference condition). The participants were asked to name the color of the ink and to ignore the written word, based on the original procedure [3]. In the fourth condition, participants were shown a page similar to the previous trial, but half of the words were enclosed in black rectangles. The participants were asked to switch between naming the color of the ink and reading the color name when the word appeared in a rectangle (Switching condition). Participants were asked to do the task as quickly as possible without making mistakes. The time to complete the task (seconds) was used as the outcome variable, with lower values indicating higher performance.

*Trail Making Test:* Executive functioning was further assessed using the D-KEFS Trail Making Test (TMT). The test has five conditions: visual scanning, number sequencing, letter sequencing, switching, and motor speed. For each condition participants’ time to complete (seconds) and number of errors were recorded. In the current study, the primary outcome was the time to complete condition 4 (switching), where the task was to connect encircled numbers and letters in alternating order switching between numbers and letters (i.e., 1-A-2-B-3-C, etc.). Because the outcome measure was time, lower scores indicate better performance.

*Verbal Fluency:* The D-KEFS Verbal Fluency test was used as a measure of letter and category fluency. The test includes three conditions: letter fluency, category fluency, and switching. In condition 1, participants orally generated as many words as possible in 60 seconds beginning with the letters F, A, and S respectively. They were instructed that proper nouns, numbers, or repeated words with a different suffix were not credited. A unit-weighted composite score (phonetic) was computed from the three letter-fluency measures (F, A, and S). In condition 2, participants orally generated as many words as possible within 60 seconds belonging to the categories ‘animals’ and ‘boys’ names, respectively. Participants were instructed to not repeat words. A composite score was computed from the two categories (semantic). In condition 3, participants orally generated as many words as possible within 60 seconds while switching between two categories: furniture and fruits. Participants were instructed to not repeat words. The total number of switches were recorded (switching). All three scores were included in the analysis (phonetic, semantic, switching).

*Ruff 2&7 selective attention test (Ruff 2&7):* The Ruff 2&7 was used as a measure of visual attention [4,5]. Participants scanned sections with three rows of numbers and letters respectively and identified the digits 2 and 7. Participants had 15 seconds per section of three rows before the test moved on to the next section. The Ruff 2&7 was scored according to the guidelines provided in the corresponding manual [6]. The primary outcomes were the Automatic Detection Speed (ADS; i.e., the sum of all hits among letter distractors) and the Controlled Search Speed (CSS; i.e., the sum of all hits among digit distractors), which were combined into a composite score. While we focus on composite scores in our study, we report the results on the individual score in the supplementary (see Table S1 for raw scores and Table S2 for results). There was a total of 20 sections of three rows where a maximum score of 600 hits could be obtained.

We included two tests from WAIS-IV, which were administered according to the manual [7].

*Digit Symbol (Coding):* To measure processing speed, the Digit Symbol Test was administered. Participants had two minutes to draw unique symbols corresponding to the numbers 1-9 using a coding key. The participants were instructed to work quickly and accurately. The outcome variable in the current study was the number of correct digit-symbol matches made within 120 seconds.

*Digit span:* The WAIS-IV Digit Span test was used to measure basic working memory and attention abilities. Participants repeated sequences of one-digit numbers in a forward and backward order respectively in two conditions. The digit spans increased with one digit every two trials. Each correct sequence is worth one point with a maximum score of 16. The task was terminated when two consecutive errors within the same digit span length were made. The primary outcome was the number of correctly recalled digit spans in backward order.

**Self-rated fatigue and mental health**

*Fatigue Visual Analogue Scale (VAS)*: Participants self-reported how tired they felt using a visual analogue scale (VAS) ranging from 0 to 100 immediately before and after completing the cognitive test battery. VAS was used as a measure of state fatigue and state fatigability. Higher values indicated worse fatigue.

In addition, participants filled out several questionnaires related to their well-being in relation to the testing session. More specifically, the following questionnaires were used to assess fatigue and mental health (anxiety and depression).

*Multidimensional Fatigue Inventory 20 (MFI-20):* The MFI-20 is a 20-item self-report questionnaire that measures five dimensions of fatigue: general fatigue, physical fatigue, reduced activity, reduced motivation, and mental fatigue [8]. Each item is scored on a 5-point scale from 1 (“Yes, that is true”) to 5 (“No, that is not true”), with a maximum score of 20 for each subscale. Higher scores indicate worse fatigue. In the present study, we focus on general, physical, and mental fatigue.

*Hospital anxiety depression scale* (HADS): this scale is a 14-item self-report screening questionnaire for depression (7 items) or anxiety (7 items; [9]). Each item is scored on a 4-point scale from 0 to 3, with higher scores indicating higher levels of depression and anxiety.

*Table S1.* Raw scores for cognitive and mental health data for patients and controls at baseline and follow-up.

|  | Patients | | | | | | | Controls | | | | |
| --- | --- | --- | --- | --- | --- | --- | --- | --- | --- | --- | --- | --- |
| Test and Measures | Baseline | | | Follow-Up | | | Baseline | | | Follow-Up | | |
|  | *N* | *M* | *SD* | *N* | *M* | *SD* | *N* | *M* | *SD* | *N* | *M* | *SD* |
| **Buschke's Selective Reminding Task** |  |  |  |  |  |  |  |  |  |  |  |  |
| Total Recall | 47 | 103 | 23 | 49 | 114 | 17 | 48 | 121 | 16 | 41 | 126 | 14 |
| Delayed Recall | 47 | 8.5 | 2.6 | 48 | 10 | 2 | 48 | 10.4 | 1.76 | 41 | 11 | 2 |
| **Color-Word Interference Test** |  |  |  |  |  |  |  |  |  |  |  |  |
| Condition 3: Interference, Seconds | 47 | 64 | 23 | 49 | 53.5 | 13.6 | 48 | 48 | 10 | 41 | 46.5 | 8.9 |
| Condition 4: Switching, Seconds | 47 | 76 | 38 | 49 | 63 | 23 | 48 | 56 | 10 | 41 | 53 | 11 |
| **Trail Making Test** |  |  |  |  |  |  |  |  |  |  |  |  |
| Condition 4: Switching, Seconds | 48 | 78.2 | 38.8 | 50 | 64.9 | 22.4 | 48 | 64.7 | 21.7 | 41 | 58.2 | 17.2 |
| **Verbal Fluency** |  |  |  |  |  |  |  |  |  |  |  |  |
| Condition 1: Phonetic | 50 | 50 | 15 | 50 | 51 | 16 | 48 | 54 | 15 | 41 | 55 | 14 |
| Condition 2: Semantic | 50 | 49 | 12 | 49 | 51 | 10 | 48 | 55 | 12 | 41 | 54 | 11 |
| Condition 3: Switching | 50 | 15 | 3 | 49 | 16 | 3 | 48 | 15 | 3 | 41 | 15 | 4 |
| **Digit Symbol (Coding)** |  |  |  |  |  |  |  |  |  |  |  |  |
| Total Score | 50 | 66 | 17 | 48 | 71 | 14 | 48 | 75 | 11 | 41 | 79 | 13 |
| **Digit Span** |  |  |  |  |  |  |  |  |  |  |  |  |
| Total Score: Backwards | 50 | 9 | 2 | 50 | 9 | 2 | 48 | 10 | 2 | 41 | 10 | 2 |
| **Ruff 2&7** |  |  |  |  |  |  |  |  |  |  |  |  |
| Automatic Detection Speed | 50 | 145 | 34 | 50 | 160 | 29 | 48 | 155 | 30 | 41 | 162 | 30 |
| Controlled Search Speed | 49 | 120 | 23 | 50 | 132.8 | 22.9 | 48 | 128 | 22 | 41 | 131.5 | 21.3 |
| **VAS** |  |  |  |  |  |  |  |  |  |  |  |  |
| Before Cognitive Testing | 49 | 47 | 22 | 50 | 44 | 23 | 48 | 27 | 19 | 41 | 26 | 19 |
| After Cognitive Testing | 47 | 67 | 22 | 50 | 58 | 27 | 48 | 32 | 20 | 41 | 31 | 19 |
| **MFI** |  |  |  |  |  |  |  |  |  |  |  |  |
| General Fatigue Score | 50 | 15.2 | 5.6 | 47 | 17.4 | 2.6 | 47 | 11.6 | 3.9 | 41 | 11.4 | 3.8 |
| Physical Fatigue Score | 50 | 15.2 | 5.2 | 47 | 15.7 | 3.9 | 47 | 9.0 | 3.8 | 41 | 8.8 | 3.9 |
| Mental Fatigue Score | 50 | 14.9 | 3.9 | 46 | 14.9 | 3.5 | 47 | 9.4 | 3.2 | 41 | 9.7 | 2.9 |
| **HADS** |  |  |  |  |  |  |  |  |  |  |  |  |
| Anxiety Score | 48 | 7.7 | 4.6 | 47 | 6.4 | 4.1 | 46 | 3.9 | 2.7 | 40 | 3.5 | 2.9 |
| Depression Score | 48 | 8.8 | 4.8 | 47 | 7.6 | 4.3 | 47 | 3.3 | 2.7 | 40 | 3.4 | 2.8 |

Note. VAS = Visual Analog Tiredness Scale; MFI = Multidimensional Fatigue Inventory, HADS = Hospital Anxiety and Depression Scale.

| *Table S2.* Results of linear mixed models for analyses involving automatic detection and controlled search speed. Baseline coefficients indicate group differences at the first time point. Significant interactions indicate differences in longitudinal changes as a function of group. | | | | | | | | | | |
| --- | --- | --- | --- | --- | --- | --- | --- | --- | --- | --- |
|  | |  | |  | | Baseline | | | Interaction | |
|  | |  | |  | Coefficient | | P-value | Coefficient | | P-value |
|  | n patients | | n controls | |  | |  |  | |  |
|  | baseline/follow-up | | baseline/follow-up | |  | |  |  | |  |
| **Ruff 2&7** |  | |  | |  | |  |  | |  |
| Automatic Detection | 50/50 | | 48/41 | | -.16 [-.57 .26] | | 0.454 | .17 [-.07 .42] | | .162 |
| Controlled Search Speed | 50/50 | | 48/41 | | .12 [-.32 .56] | | 0.596 | -.01 [-.44 .42] | | .950 |

*Table S3*. Spearman’s rank correlation between demographics or mental health variables (baseline) and changes in cognitive tasks

(follow-up minus baseline) in patients.

|  | Delayed Recall (SRT) | | Interference (CWIT) | | Switching (CWIT) | |
| --- | --- | --- | --- | --- | --- | --- |
|  | Spearman's r | p-value | Spearman's r | p-value | Spearman's r | p-value |
| Age | -0.08 | 0.583 | 0.16 | 0.295 | 0.05 | 0.760 |
| Sex | -0.19 | 0.214 | 0.31^*^ | 0.039 | 0.15 | 0.335 |
| Education | 0.19 | 0.210 | -0.09 | 0.515 | -0.13 | 0.401 |
| History of Burnout | -0.17 | 0.272 | 0.37^*^ | 0.012 | 0.24 | 0.116 |
| History of Depression | 0.02 | 0.912 | -0.02 | 0.892 | 0.24 | 0.105 |
| HADS - Anxiety | -0.19 | 0.226 | -0.03 | 0.864 | -0.16 | 0.302 |
| HADS - Depression | -0.09 | 0.582 | -0.21 | 0.176 | -0.34^*^ | 0.025 |
| VAS-Before | 0.01 | 0.936 | -0.14 | 0.356 | -0.16 | 0.310 |
| VAS-After | 0.14 | 0.377 | -0.11 | 0.469 | -0.17 | 0.281 |
| MFI-General Fatigue | 0.15 | 0.336 | -0.21 | 0.165 | -0.22 | 0.135 |
| MFI-Physical Fatigue | 0.14 | 0.375 | -0.09 | 0.544 | -0.09 | 0.568 |
| MFI-Mental Fatigue | -0,01 | 0.952 | -0.16 | 0.280 | -0.14 | 0.339 |

Note. SRT = Buschke’s selective reminding task, CWIT = Color-Word Interference Test, HADS = Hospital anxiety depression scale,

VAS = Fatigue Visual Analogue Scale, MFI = Multidimensional Fatigue Inventory. * p < .05

**References**

1. Buschke H. Selective reminding for analysis of memory and learning. J Verbal Learning Verbal Behav. 1973;12(5):543–50. doi10.1016/S0022-5371(73)80034-9
2. Delis DC, Kaplan E, Kramer JH. Delis-Kaplan Executive Function System. PsycTESTS Dataset [Internet]. 2012 Oct 8 [cited 2025 Aug 25]; Available from: https://doi.apa.org/doi/10.1037/t15082-000
3. Stroop JR. Studies of interference in serial verbal reactions. J Exp Psychol. 1935;18(6):643–662. doi.org/10.1037/h0054651
4. Ruff RM, Niemann H, Allen CC, Farrow CE, Wylie T. The Ruff 2 and 7 Selective Attention Test: a neuropsychological application. Percept Mot Skills. 1992;75(3 Pt 2):1311–9. doi/pdf/10.2466/pms.1992.75.3f.1311
5. Ruff RM, Evans RW, Light RH. Automatic detection vs controlled search: A paper-and-pencil approach. Vol. 62, Perceptual and Motor Skills. US: Perceptual & Motor Skills; 1986. p. 407–16. doi:10.2466/pms.1986.62.2.407
6. Ruff RM, Allen CC. Ruff 2 & 7 Selective Attention Test. Odessa, FL: Psychological Assessment Resources; 1996.
7. Wechsler D. Wechsler Adult Intelligence Scale-Fourth Edition. 2008.
8. Smets EM, Garssen B, Bonke B, De Haes JC. The Multidimensional Fatigue Inventory (MFI) psychometric qualities of an instrument to assess fatigue. J Psychosom Res. 1995 Apr;39(3):315–25. doi:10.1016/0022-3999(94)00125-o
9. Zigmond AS, Snaith RP. The hospital anxiety and depression scale. Acta Psychiatr Scand. 1983;67(6):361–370. doi:10.1111/j.1600-0447.1983.tb09716.x
